# X-ray Imaging and Computed Tomography for the Identification of Geometry and Construction Elements in the Structure of Old Violins

**DOI:** 10.3390/ma14205926

**Published:** 2021-10-09

**Authors:** Mariana Domnica Stanciu, Mircea Mihălcică, Florin Dinulică, Alina Maria Nauncef, Robert Purdoiu, Radu Lăcătuș, Ghiorghe Vasile Gliga

**Affiliations:** 1 Faculty of Mechanical Engineering, Transilvania University of Brasov, B-dul Eroilor 29, 500360 Brasov, Romania; 2 Russian Academy of Natural Sciences Sivtsev Vrazhek, 29/16, 119002 Moscow, Russia; 3 Departament of Forest Engineering, Forest Management Planning and Terrestrial Measurements, Transilvania University of Brașov, 500123 Brașov, Romania; dinulica@unitbv.ro; 4 Faculty of Music, Transilvania University of Brașov, 500360 Brașov, Romania; a_nauncef@unitbv.ro; 5 Faculty of Veterinary Medicine Cluj Napoca, University of Agricultural Sciences and Veterinary Medicine Cluj-Napoca, Calea Mănăștur 3-5, 400374 Cluj-Napoca, Romania; robert.purdoiu@usamvcluj.ro (R.P.); radu.lacatus@usamvcluj.ro (R.L.); 6 Faculty of Furniture Design and Wood Engineering, Transilvania University of Brasov, B-dul Eroilor 29, 500360 Brasov, Romania; vasile.gliga@unitbv.ro; 7 S.C. Gliga Musical Instruments S.A., str. Pandurilor 120, 545430 Mureș, Romania

**Keywords:** old violin, X-ray imaging, computed tomography, resonance wood, constructive elements

## Abstract

Numerous studies on heritage violins have shown that there are a number of factors that contribute to the acoustic quality of old violins. Among them are the geometric shape of the violin, the thickness of the tiles, the arching of the tiles, the dimensions and position of the bass bar, the size and position of the acoustic holes. Thus, the paper aims to compare the structural and constructive elements of old violins made in various famous violin workshops (Stainer, Klotz, Leeb, Babos Bela), using nondestructive and noncontact techniques based on image analysis. The violins that were studied date from 1716 to 1920, being in good condition, most of them being used by artists from the Brașov Philharmonic of Romania. In the first stage of the study, the violins were optically analyzed and scanned to identify the structure of the resonant wood, using the WinDENDRO Density 2007 program. X-ray imaging and computed tomography (CT) were also used. Combining the types of analyses, capitalizing on the expertise of violin producers and the knowledge of researchers in the field, valuable data on the geometric and constructive characteristics of old violins were extracted.

## 1. Introduction

It is unanimously recognized that the queen of stringed musical instruments is the violin, an instrument whose shape, size and materials have reached the highest performance of musical sounds, through the ancient and established luthiers Andrea Amati (1505–1578), Giuseppe Guarneri del Gesù (1698–1744) and Antonio Stradivari (1644–1737). The current shape of the violin was established by Andrea Amati (1505–1578); over time, violin makers brought only small changes into the constructive elements, almost imperceptible to an ordinary visual analysis. Many of these musical instruments are rare examples of high artistic mastery and are still used as a reference in the contemporary manufacture of violins [1,2,3]. In addition to the consecrated violin makers mentioned above, violin making workshops have been developed in other regions of Europe, through violin makers who completed their apprenticeship in Italian violin makers’ workshops and who imprinted the specifics of the area on their violin models.

Therefore, Jacobus Stainer (1619–1683) was the most famous luthier of the Austrian-German schools, being born in Absam (Tyrol). He is supposed to have been the disciple of Nicolò Amati of Cremona, although the manuscripts and historical evidence are not complete enough to justify this assumption. In any case, his work, the oldest of which dates from the 1630s, bears a strong resemblance to that of Amati. Stainer eventually settled in his hometown of Absam in 1656, where he began producing some of his best instruments, which appear to be inspired by Amati’s models. During this period, Stainer created his own style, producing exceptional instruments that rivaled or even surpassed the works of his Cremonese contemporaries of the seventeenth century. As specific constructive features, we can notice that the arching of the front plates is higher than that of the rear plates; the growth is maintained up to half the length; and the finish used is yellow, with a shade of pale rose [4,5,6]. The resonant bar is terminated at its two ends by bevels that extend on the sound plate to which it is glued, being placed under the G string of the instrument.

Joseph Thomas Klotz (1743–1819) was the son of Sebastian Klotz, one of Stainer’s best disciples and successors, and he had his workshop in Mittenwald, Germany. Historians believe that this artist built the violins according to his father’s system, but he knew the qualities of wood better; therefore, his instruments were superior in tone, but inferior in the finish (in their initial, original state).

Johann Georg (II) Leeb (1740–1813) was the son of Johann Georg Leeb (I), both of whom marked the Hungarian violin school. Johann Georg Leeb worked in Presburg (now Bratislava). Leeb violin designs are quite distinct, with flat arches with a certain influence of the violinist Carlo Bergonzi (1683–1747). Johann G. (II) Leeb was a prolific manufacturer and produced violins of different quality classes, using different materials. He was succeeded by his son Johann Georg (III), born in 1779.

**Figure 1:**
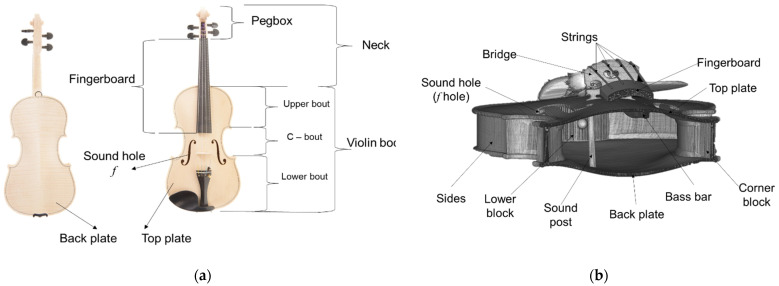
The constructive elements of the violin: (**a**) the front view of the violin; (**b**) cross section through the violin.

Babos Bela was a representative of the Hungarian violin school from the beginning of the 20th century [5,6]. In Romania, in the well-known city of violins, Reghin, the art of violin making developed to a level of qualitative and aesthetic perfection, with numerous violin workshops, due to the high quality of wood existing in the area known in antiquity as the Italian valley. At first glance, the constructive form of the violin and the wood in its structure have been preserved over the centuries, but there are constructive details that differentiate the style of the violinists and even the acoustics of the instrument. The constructive elements of a violin have both a functional and an aesthetic role. Thus, the body of the violin, composed of the upper plate, the back plate, straps and counter-straps, has the acoustic role of amplifying the musical sounds emitted during the movement of the bow over the strings. For reasons of mechanical strength, the body of the violin also contains constructive elements that fix the two plates (by means of straps, counter-straps, hubs and corners) and elements that support and fix the neck of the violin (Figure 1). The plates have a spatially curved shape in both the longitudinal and transverse directions. Their thickness varies from the center (the area between the holes f) to the edges. From the wood species point of view, a selected (for the structural characteristics) softwood (spruce—Picea Abies L. Karst) is used for the top plates of the violins (as well as of all stringed instruments) and curly maple wood (Acer pseudoplatanus L.) is used for the back plates. Previous research has shown that old violins emit much clearer, brighter, louder sounds than new violins. Over time, the determining factors for this aspect have been analyzed, starting from the structural quality of the wood, moisture content, wood ageing, slab geometry (thickness/arching), finishes, constructive elements of the violins (stern, position and shape of acoustic holes, sounding bar, gag) and string quality [7,8,9,10,11]. It has not yet been possible to detect the predominant factor, thus the research remains open. The non-invasive structural analysis of historical musical instruments is a fundamental tool for defining restoration and conservation protocols, as well as for the study of ancient manufacturing techniques and acoustic analysis related to this class of cultural objects. The importance and value of typical bowed string instruments, on the other hand, requires a non-destructive approach with strict environmental control, fast acquisition times and high spatial resolution [12,13,14,15,16].

Through various non-invasive and non-destructive modern methods and techniques, numerous researchers have performed structural assessments of musical instruments, highlighting the richness of details, characterizing their internal structure, identifying defects, assessing the thickness of structural elements of wood and its density and conducting a dendrochronological investigation of historical violins [16,17,18,19].

**Figure 2:**
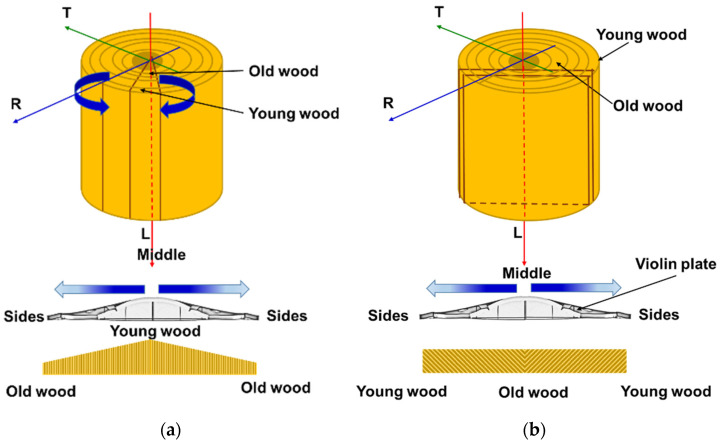
The main types of wood cuts for the violin: (**a**) quarter sawn; (**b**) live sawn.

From a constructive and technological point of view, the top plates of a violin are obtained from two halves, cut radially longitudinally from the logs (as in Figure 2), which, after their natural drying, are conditioned in a drying chamber up to a moisture content of 6–8%. Then, the pairs boards are glued lengthwise, obtaining a plate with an anatomical structure of the wood symmetrical to the median longitudinal axis.

Most of the wood that is used in a violin’s plates construction is cut on the quarter. Nyman, 1975 [20], highlights the fact that most Cremonese violins have the wood cut into quarters (Figure 2a), compared to the style of Brescians violin makers who use live sawn, which has an end grain with growth rings of 0–90 degrees to the surface (Figure 2b). In order to obtain the arching of the plates by roughing, the initial thickness of the semi-finished products cut from logs is higher toward the middle of the plate and smaller toward the sides. Thus, the age of the wood is chronologically higher towards the outside of the violin and lower towards the inside.

The novelty of the paper consists in the comparative analysis of six violins (from XVIII–XX centuries) belonging to well-known violin schools, Stainer, Klotz, Leeb, Bela and Gliga, in order to identify their constructive particularities and the anatomical characteristics of the wood in their structure. All this information is a scientifically valuable database, especially since most studies thus far have focused on the heritage violins of the great Italian luthiers.

## 2. Materials and Methods

### 2.1. Studied Structures

**Figure 3:**
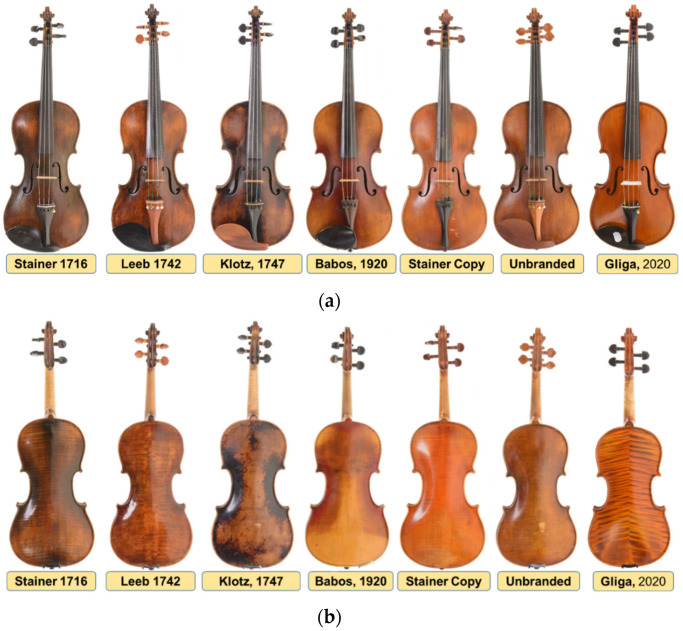
Old violins analyzed using non-invasive and non-destructive methods: (**a**) front view of studied violins; (**b**) back view of violins.

In this study, six old violins and a current one were analyzed, five of them with labels containing information on the date of manufacture and belonging to a violin school: violin Jacobus Stainer, 1716; violin Johann Georg Leeb, 1742; violin Joseph Klotz, 1747; violin Babos Bela, 1920; violin Gliga Vasile Ghiorghe, 2020, and two without a label. For one of the unlabeled, the history is known (the fact that it is a copy of Jacobus Stainer coded “Jacobus Stainer Copy”), and for the other violin (coded “Unbranded”), the origin and whether it belonged to a certain school of violinists are unknown (Figure 3).

Figure 3 shows the seven violins studied, with images of both the faces of the violins (Figure 3a) and the back of the violins (Figure 3b). All the violins studied are constructively intact, being used in musical activities by their owners. For this reason, the methods of analysis of the constructive elements were chosen so as not to damage or harm the violins in any way. Additionally, the color of the finish of the violins and the quality of the surface in terms of clarity of wood structure at the time of investigations can be seen in Figure 3. Aspects related to the color tones, the type of finishes and the thickness of the penetration of the wood finish were studied by [3,11,12]. It was found that frequently used and aged instruments show a pattern of wear due to the degradation of the varnish after extensive manipulation and weighing by the violinist, which makes it difficult to analyze the structure of the wood [20,21,22].

### 2.2. Methods

#### 2.2.1. Wood Structure Data Acquisition

**Figure 4:**
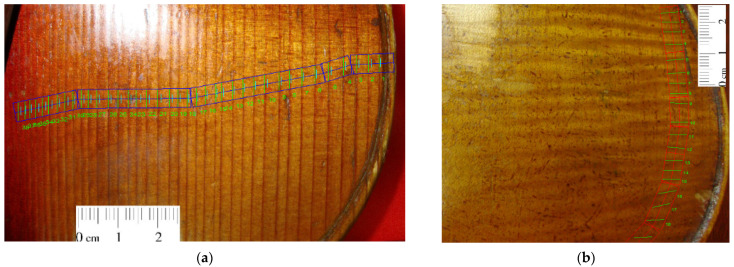
The constructive principle of violin boards for obtaining the anatomical symmetry of the wood structure: (**a**) measurement of annual rings, early wood–late wood with Windendro system 2007 in case of violin top plate; (**b**) measurement of wavelength with Windendro system 2007 in case of violin back plate.

Evaluation of the anatomical features of the wood in the construction of the top and back plates of old violins was performed using a WinDENDRO Density image analysis system (Régent Instruments Inc., Québec City, QC, Canada, 2007) from the Department of Forest Engineering, Forest Management Planning and Terrestrial Measurements, Transilvania University of Brașov, Romania (Figure 4a,b). The characteristics of the annual rings were measured in terms of the width of the annual rings denoted TRW, the width of the early wood EWW, the width of the late wood LWW and the wavelength of the curly fiber of maple (wavelength CWL) according to the method presented in previous studies [23,24,25]. The annual rings were measured in two or three directions, depending on the objective local difficulties in identifying the contour of the rings, especially for old violins, starting from the edge of the sides to the welding line of the face halves (Figure 4a,b). For spruce boards in the structure of violin top plates, the width of early wood and late wood was measured, the wood structure made it possible to take these data, while for maple boards in the construction of the back of the violin, only the width of each ring was measured. For verification, the resulting series of face rings were cross-dated to each other. Cross-dating was conducted within the same software, adopting a threshold of 0.60 for the Gleichläufigkeit correlation coefficient [26]. All measurements were performed without removing the metal strings and other accessories, as requested by the violins’ owners.

#### 2.2.2. X-ray Imaging

**Figure 5:**
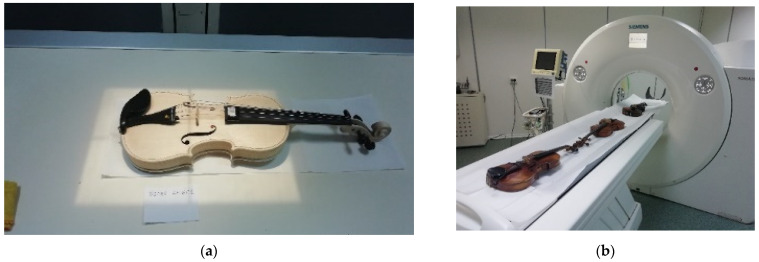
Old violins analyzed using non-invasive and non-destructive methods: (**a**) X-ray of the violin; (**b**) Computed Tomography analysis of old violins.

In order to determine the shape and geometry of the violins, the samples were exposed to X-ray radiography at the Laboratory of Radiology and Medical Imaging, Faculty of Veterinary Medicine of Cluj-Napoca (Figure 5a). The X-ray exposures were made using a fixed radiographic device TEMCO Grx-01 (K&S Röntgenwerk Bochum GmbH&Co KG, Bochum, Germany). The exposures were made dorsoventrally, the field of view being set to cover the violin body. The parameters used to obtain the images were 50–56 kV and 13–20 mAs. The images were acquired using a DR Flat Panel detector Reyance Xmaru 1717SGC/SCC (Reyance Inc., Hwaseong-si, Gyeonggi-do, South Korea) and Xmaru VetView (Reyance Inc., Hwaseong-si, Gyeonggi-do, Korea) acquisition software.

#### 2.2.3. Computed Tomography

The violins were investigated using computer tomography (denoted CT) in order to analyze the constructive elements, thicknesses and arching of the old violins (Figure 5b). The CT examinations were performed on a Siemens Somatom Scope (Siemens, Erlangen, Germany) helical CT device with 16 slices. The scans were performed using a bone reconstruction kernel. The images acquisition was conducted at 2 mm/slice and the reconstruction was performed at 0.75 mm/slice. For each violin, two axial scans were performed, one for the violin body and the second for the violin neck. The scan parameters were Nominal Total Collimation Width: 9.6 mm, Pitch Factor: 0.8 ratio, KVP: 130 kV, X-ray Tube Current: 96 mA, Exposure: 120 mA, Exposure Time per Rotation: 1 s, 512 × 512 Matrix. The images, both for X-ray and CT scan, were acquired in DICOM format; reading and post-processing of the DICOM files was performed using 3DNET PACS software and Horos DICOM viewer.

#### 2.2.4. Data Processing

The raw data were processed, calculating the early wood proportion (EWP) and the latewood proportion (LWP). In order to assess the regularity of the rings, the following method of calculating the regularity index RI, recommended by Dinulică et al., 2015, [23] for wood, was adopted for the construction of violins:

(1) $$RI=\frac{\max\left(TRW_{i}\right)-\min\left(TRW_{i}\right)}{\max\left(TRW_{i}\right)},$$

where *i* is a ring from the middle series of the front or back plate ($i=\bar{1\ldots n\;})$ and *n* is the length of the series.

Then, the data were imported and processed in STATISTICS 8.0 (StatSoft 2007), following Zar’s instructions (1974) [27]. To start, the variability of the experimental data was explored, and the normality was verified with the Shapiro–Wilk test. Then, the significance of the differences between the violins regarding the size of the wood structure variables was tested.

## 3. Results and Discussions

### 3.1. The Anatomical Analysis of Wood from the Construction of Old Violins

A total of 2641 front rings and 970 back rings were measured and, in Table 1 the average values and standard deviation of the main characteristics of the annual rings measured on the top and back plates of the violins are summarized.

**Figure 6:**
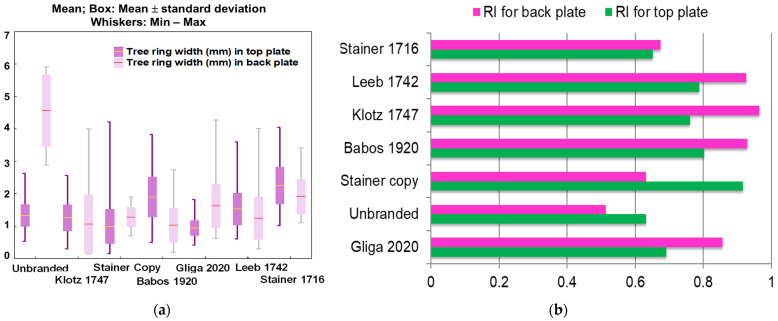
Comparison between anatomical descriptors of wood from old violins structures: (**a**) The variation of the annual ring width in case of top and back plates of studied violins; (**b**) The regularity level of the annual rings in the structure of the sound box for the analyzed violins.

**Figure 7:**
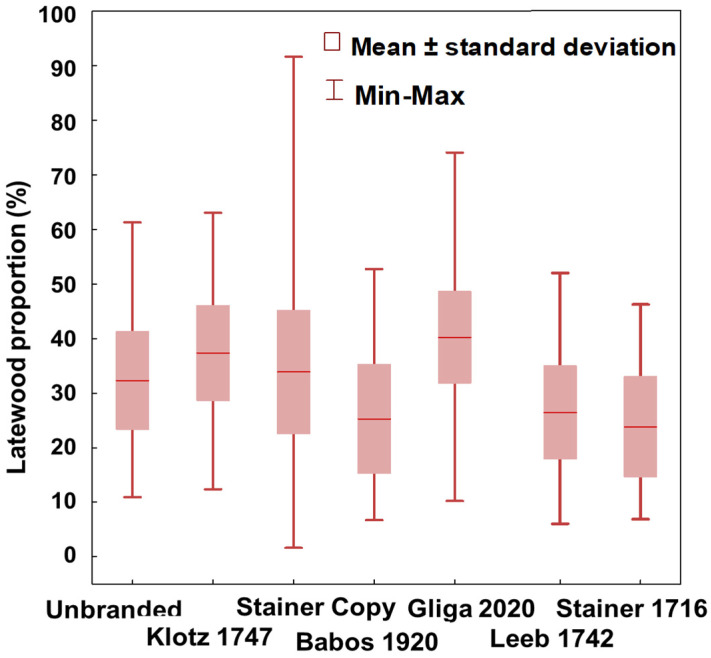
The variation of the proportion of late wood of top plates in the analyzed violins.

From a statistical perspective, the measured characteristics of the wood structure of the violin sound box are continuous variables. They are not compatible with the normal law (*W from Shapiro–Wilk = 0.886–0.992*, *p < 0.001*), and the non-parametric Kruskal–Wallis test shows that the analyzed violins differ from each other at a very significant level in terms of all the structural characteristics (*H = 257–1272*, *p < 0.001*). Therefore, each violin has its structural personality, as can be seen in Figure 6 and Figure 7. In three of the violins analyzed, the annual rings in the back plate structure are considerably finer than those in the top plate structure (Babos 1920, Leeb 1742, Stainer 1716), while in the other three violins, the annual rings of top plate are much finer than those in the back (Unbranded, Stainer Copy, Gliga 2020); for the Klotz 1747 violin, the rings have close widths in the two plates of the sound box (Figure 6a). In 40% of cases, the regularity index of the width RI (Figure 6b) is within the limits specified by Rocaboy et al. (1990) [28] for the resonant wood (*RI* *≤ 0.700*). It is known that the higher the RI value is, the lower the regularity of the rings is. In most violins, there are big differences between the top and back plates regarding the regularity of the rings. The rings of the spruce wood (top) are usually more regular than those of the back (maple wood) (Figure 6b). The width of the early wood in the composition of the annual ring is directly proportional to the width of the annual ring (*Spearman R rank order correlation: 0.975*, *p < 0.001*). Additionally, the width of the late wood depends to a large extent on the width of the growth ring (*Spearman R: 0.651*, *p < 0.001*). On average, late wood accounts for a third, and early wood the other two-thirds of the annual ring width. The proportions of the two components of the annual ring show a moderate level of variability (coefficient of variation: 16 and 32%, respectively) (Figure 7).

Of all the old violins investigated, 66% of the recorded values of the proportion of late wood exceed the reference level of 25% mentioned for the resonant spruce by Bucur, 2006 [29]. There are violins in which the central tendency of the late wood increases to 40% of the width of the ring, such as Klotz 1747 and Gliga 2020 (Figure 7).

**Figure 8:**
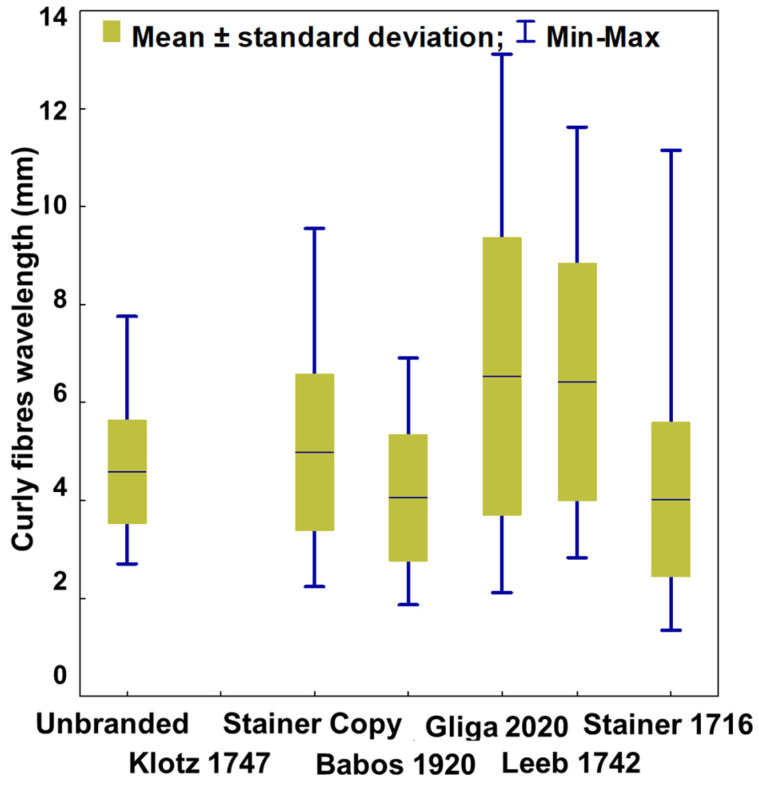
The variation of the curly fibers’ wavelength from maple back plates of analyzed violins.

It is not excluded that the result may be influenced by violin finishing techniques, which may have led to an overestimation of the width of the wood late in the imaging analysis. However, we must also take into account the fact that high values of LWP are recorded in narrow rings (the correlation coefficient between the width of the ring and the proportion of late wood is −*0.623*, *p < 0.001*) and rings that abound in the violins analyzed. Specifically, 38% of the total number of rings measured is less than 1 mm wide. Regarding the characteristics of curly maple wood, the wavelength of the curly fiber gravitates around 4.4 mm. The smallest value (1.35 mm) was measured on the Stainer 1716 violin, and the highest (13.11 mm) on the Gliga 2020 violin. The differences between the violins are noticeable, some have tightly created fibers, others are wide (Figure 8). It is a tendency to associate the wavelength with certain values of the annual ring width; respectively, the dense fiber appears especially in maple wood with wider rings (*Spearman R rank order correlation: −0.156*, *p = 0.04*).

### 3.2. X-ray Radiography of Heritage Violins

**Figure 9:**
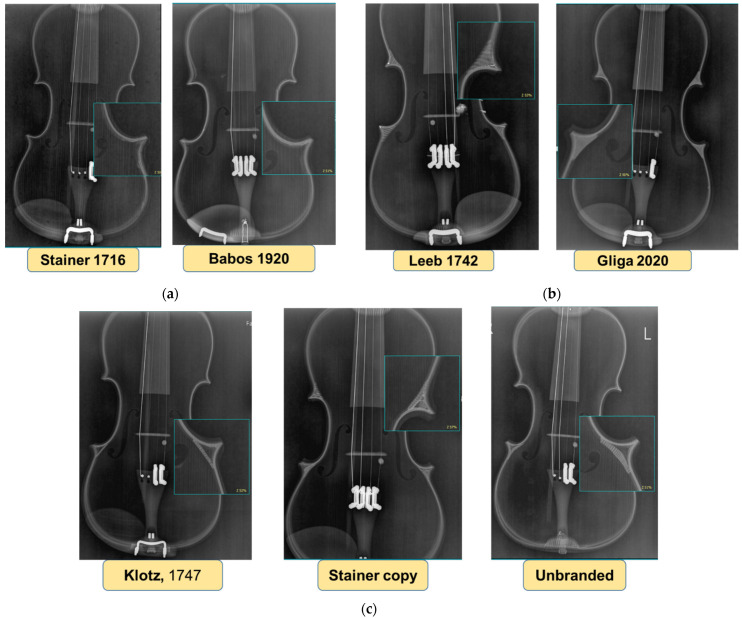
The constructive shape of corners visible on X-ray radiation: (**a**) Violins without corners; (**b**) Violins with solid wood corners; (**c**) Violins with softwood slates.

Through the X-ray analyses within the Laboratory of Imaging Analysis and Radiography of the Faculty of Veterinary Medicine, USAMV Cluj-Napoca, it was possible to identify some constructive elements specific to the violin schools to which the violins belong. The characteristics that can be observed in X-ray radiography are the macroscopic elements of the structure, the poor resolution being, on the one hand, due to the relatively low sensitivity of X-rays to wood and, on the other hand, due to the large sample size [30]. Thus, one of the obvious constructive elements in the X-ray analysis is the corners, which have the role of strengthening the intersection between the curves of the violin as a result of changing the curvature radius, as well as increasing the gluing surface between the front plate, back plate and straps (Figure 9). As can be seen in Figure 9, the investigated violins can be grouped into the following three classes in terms of the constructive shape of the corners: violins without a corner on the inside (Stainer, 1716; Babos 1920—Figure 9a); another category of violins is the one in which the corners are stiffened with solid wood corners, cut according to the inner shape of the corners, obtaining a continuous contour inside the violin body (Figure 9b); and finally, violins with softwood slates (Klotz, 1747, the Stainer copy and the “unbranded” violin), noting that the corner reinforcing slates on the Klotz 1747 violin are found only at the corners between the central curvature and the lower curvature (Figure 9c). A few ways of joining the corners are presented in the literature [4,5].

### 3.3. Imaging Analysis of Old Violins Using CT Scanning

**Figure 10:**
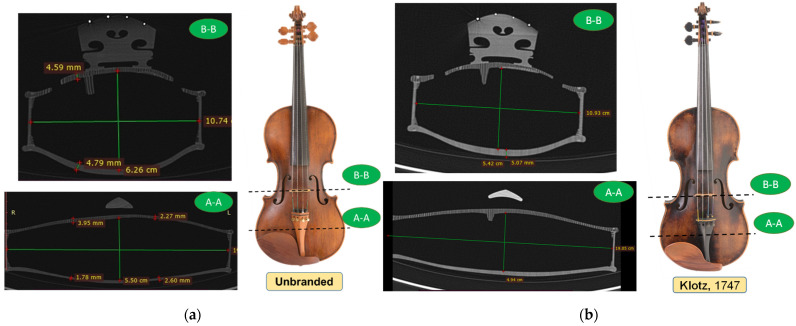
CT images analysis: (**a**) the Unbranded violin; (**b**) the “Klotz, 1747” violin.

Information on the thickness of the violin plates, the curvature of the plates, the shape of the sound bar, the dimensions of the old violins and aspects regarding the integrity or the degree of damage of the violins, all this was obtained using computed tomography of the studied violins. Computed tomography comprises a set of axial 2D images. The data volume can be reformatted and reorganized into 3D images, with the advantage of obtaining a contrast approximately 16 times higher than X-ray radiography, in order to identify some constructive elements (shapes/dimensions) inaccessible to the “naked eye”. In Figure 10a,b, two cross sections are presented: one through the “unbranded” violin (Figure 10a), and one through the “Klotz, 1747” violin (Figure 10b).

**Figure 11:**
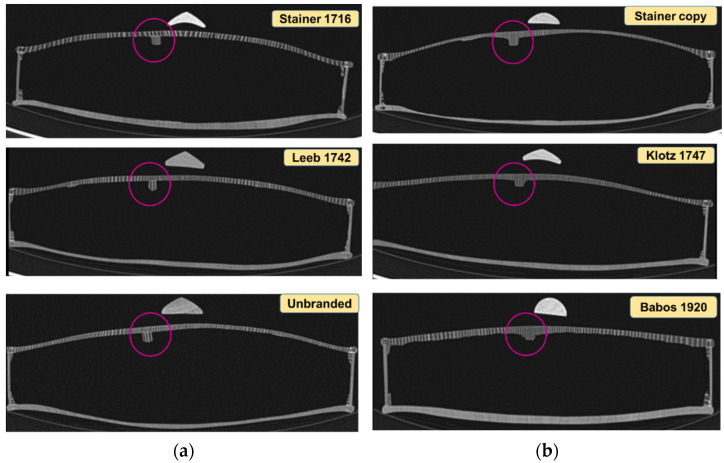
Cross section through CT-scanned violins: (**a**) violins with the applied resonance bar; (**b**) violins with the resonant bar processed from the top plate thickness.

Similarly, the other violins were analyzed, obtaining the dimensions of the violins in different sections, the thickness of the plates and the radius of curvature. From a constructive point of view, an interesting detail is observed in the way the resonance bar was made. The Stainer, 1716, Leeb 1742 and “unbranded” violins show the bass bar applied on the top plate (Figure 11a), compared to the violins “Stainer copy”, Klotz 1747 and Bela 1920, in which the resonant bar was made by roughing the front plate, with a volumetric element on the inside of the top plate (Figure 11b).

Additionally, the arching of the violin plates and the thickness of the plates play an important role in the acoustics of the musical instrument, giving the violins the signature modes, as [31] calls them, these modes being cavity modes (A0, A1), corpus modes (CBR or C bouts rhomboidal) and main body resonance (B1+ and B1−).

**Figure 12:**
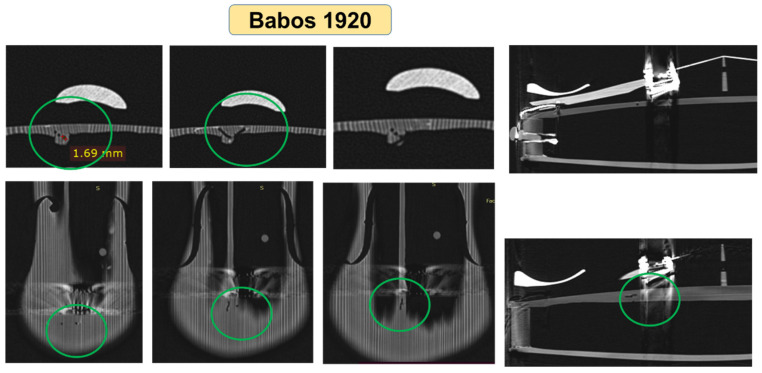
The trajectory of voids produced by coleoptera in case of the violin “Babos, 1920”.

**Figure 13:**
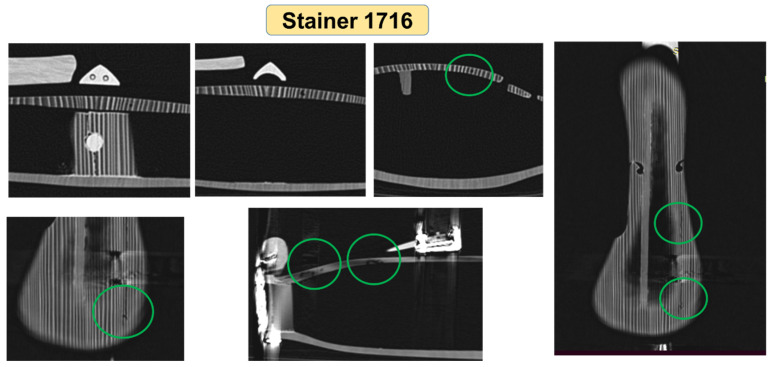
The trajectory of voids produced by coleoptera in case of the violin “Stainer, 1716”.

Another constructive characteristic is the one related to the composition of the back plates: Unbranded and Klotz violins have a back plate made of a single wooden board, while the other violins have a back plate composed of two halves with the symmetrical and quasi-symmetrical structure of annual rings. This aspect can be observed even with the naked eye by visual analysis of the violin, but it is also confirmed by the cross-sectional views obtained on the computed tomography. The CT images offer the possibility to clearly distinguish the differences between the two wood species used for the front (spruce) and back (maple) boards, as well as eventual interventions/repairs performed on the violins, the degree of wood wear and biological attacks of the wood, as can be seen in Figure 12, in the case of the Babos 1920 violin, and Figure 13, for the Stainer 1716 violin. In the highlighted areas in Figure 12, the trajectories of the voids produced by larvae of coleoptera can be observed, the color contrast and shape of the voids being specific to the biological attack. Taking into account the way the violin plates were assembled, where the young wood is found in the joint area, we assume that the holes observed at CT were produced by *Anobium pertinax or Anobium punctatum*, which laid eggs under the rhytidome, and the larvae bear irregular galleries of maximum 3 mm and filled them with sawdust. The wood can be attacked inside without being noticed on the outside [32,33].

## 4. Conclusions

The study presents the non-invasive spectroscopic approaches related to anatomical patterns of wood from ancient and modern violin plates, as well as the morphological and geometrical features, such as the shape of the body, the arching, the corners and the f-holes. Integrating and comparing the results, it was possible to characterize the studied violin, as can be seen in Table 2. Finally, it can be appreciated that the integration of imaging techniques with information on wood processing and its properties provides a useful database for luthiers and musicians, and in perspective, the authors of this paper aim to both date unknown violins (Unbranded and Stainer copy) and conduct acoustic analysis on these violins, compared to the Stradivarius Elder Voicu 1702 violin, which is part of Romania’s cultural heritage.

In further work, the correlation between the anatomical structure of wood, especially the values of the indicators for characterizing the symmetry and the frequencies spectra, dominant frequencies, quality factor and damping of old and new violins from A dynamic test will be presented. For each violin, the signature mode will be identified and quantified in terms of eigenvalues.

## Figures and Tables

**Table 1:** The anatomical features of spruce and maple wood from top and back plates of studied violins.

Variables	Studied Violins						
Average Values/STDV	Stainer 1716	Leeb 1742	Klotz 1747	Babos 1920	Stainer Copy	Unbranded	Gliga 2020
Top Plate (Spruce Wood)							
Annual rings widths (mm)	2.247	1.530	1.251	1.891	0.985	1.327	0.940
	0.567	0.490	0.403	0.612	0.527	0.336	0.234
Early wood width (mm)	1.676	1.148	0.792	1.449	0.689	0.907	0.568
	0.518	0.467	0.304	0.601	0.450	0.293	0.190
Latewood width (mm)	0.496	0.382	0.459	0.442	0.300	0.420	0.372
	0.178	0.122	0.162	0.158	0.118	0.130	0.100
Early wood proportion (%)	76.184	73.564	62.635	74.379	66.127	67.689	59.766
	9.152	8.507	8.700	9.942	11.286	8.921	8.388
Latewood proportion (%)	23.816	26.436	37.365	25.203	33.873	32.311	40.234
	9.152	8.507	8.700	9.942	11.286	8.921	8.388
Back Plate (Maple Wood)							
Annual rings widths (mm)	1.908	1.246	1.063	1.026	1.277	4.563	1.623
	0.531	0.658	0.902	0.527	0.297	1.105	0.666
Wavelength (mm)	4.021	6.421	NA	3.946	4.984	4.585	6.731
	1.577	2.422	NA	1.256	1.589	1.057	3.371

**Table 2:** The features of studied violins.

Features	Studied Violins						
	Stainer 1716	Leeb 1742	Klotz 1747	Babos 1920	Stainer Copy	Unbranded	Gliga 2020
Types of wood cuts for the violin	Two pieces	Two pieces	One piece	Two pieces	Two pieces	Two pieces	Two pieces
Annual rings width	Wide	Narrow	Narrow	Wide	Narrow	Narrow	Narrow
Regularity of annual rings	Medium	Weak	Good	Medium	Weak	High	High
Symmetry of top plate	Medium	Weak	Medium	Weak	Weak	Medium	High
Types of wood cuts for the violin	Two pieces	Two pieces	One piece	Two pieces	Two pieces	Two pieces	Two pieces
Annual rings width	Wide	Narrow	Medium	Narrow	Narrow	Wide	Wide
Wavelength	Medium curly fibers	Low curly fibers	No curly fiber	Low curly fibers	Medium curly fibers	Medium curly fibers	High curly fibers
Regularity of annual rings	Medium	Weak	Weak	Weak	Medium	Medium	Weak
Constructive Elements							
Type of bass bar	Applied	Applied	From top plate	From top plate	From top plate	Applied	Applied
Type of corner	No corners	Solid wood	Softwood slats	No corners	Softwood slats	Softwood slats	Solid wood
Coleoptera voids	Yes	No	No	Yes	No	No	No

## Data Availability

The data presented in this study are available on request from the corresponding author.
